# Aspects of communication in medical life. Doctor-patient communication: differentiation and customization

**Published:** 2017

**Authors:** D Borţun, CS Matei

**Affiliations:** * National School of Political and Administrative Studies, Bucharest, Romania; ** ”Titu Maiorescu” University, Bucharest, Romania

**Keywords:** culture, efficient communication, communicational culture, communicational culture of the medical professionals, patient-orientation

## Abstract

One of the weaknesses of the Romanian medical system is the absence of the communicational culture. This absence is felt at all levels of the healthcare system: doctor-patient relationship, doctor-patient’s relatives relationship, labor relations within the medical teams and units, the management of the large hospitals and of the medical institutions from the public administration system and last, but not least, the relationships of these units and institutions with the public opinion and, particularly, with the stakeholders.

This paper tackled with some of the principles and values that underlie an efficient communication, the default of which was felt in various domains of the Romanian medical life. They were analyzed from the perspective of the Romanian and international literature and the conclusions drawn might inspire proposals for the improvement of the medical education as well as for the professional development of the Romanian doctors.

**Dimensions of the communicational culture**

Cultural studies, a field developed in the last decades, provides us with hundreds of definitions of culture, but our approach here shall revolve around a synthetic definition which we formulated [**1**], based on those encountered in the specialized literature: set of principles, values and symbols, rules, rituals and institutions that mediate the relations between the members of a community, between community and each of its members, between community and other communities, as well as the relation of the community with the cosmic environment (natural environment in general and transcendence). As such, the notion of “communicational culture” refers to those principles, values and symbols, rules, rituals and institutions related to communication. It is these landmarks underlying the communicational culture that render both the “communicational thinking” [**9**] and, consequently, the competitive communicational behavior possible. If we give the concept of “community” the meaning of professional community and we apply it to the Romanian medical body, we may speak of the communicational culture of the Romanian medical body. 

Communication is part of our life, it is essential for living and working, embodying a simple fact: by practicing it, a person tries to create a community with other individuals through which information, representations, ideas, values and attitudes can be disseminated. However, communication is more than issuing sounds and words, it represents, at the same time, thinking and knowing. First of all, language is an instrument of thinking and of knowledge (including self-knowledge) and then an instrument of communication. In terms of actions and situations, of the variety of forms and levels of human relations that it mediates, communication may capture a multitude of meanings, with an infinity of nuances. The organizational communication is based on the inter-group communication and the latter, in its turn, functions based on the interpersonal one.

What at a personal level is a psychic element, at an interpersonal level it becomes support of inter-psychical relations. As such, lines of correspondence are established: perception and thinking -> epistemic relations; language -> communication relations; affectivity -> preferential relations; aptitudes -> functional relations; typological and temperamental traits -> domination relations. In their turn, inter-psychical relations crystallize at the level of the small group as structures or networks: perceptive, communicational, preferential, occupational, structures of power and of leadership. At the level of the large groups, such networks become the support of some mass phenomena: rumors, fashion, opinions, tastes, attitudes, and collective behaviors. From one level to another, the psychical values conveyed by an individual boost and metamorphose, but their identity remains intact (they do not become anonymous). What really changes is the functioning arrangement: from strongly informal (highly intimate interpersonal relations) to more and more formal and institutionalized relations (up to the level of an organization or of mass activities). The isoformism between the different levels of the psychic, personal, interpersonal, group, organizational, and societal organization is broadly analyzed elsewhere [**2**]. 

Being a complex process, communication also encapsulates, beyond the structural side, other aspects of the organizations’ existence, namely technical, educational, psychological, cultural aspects, etc. In order to achieve an efficient communication, we have to observe some rules, which, albeit seemingly simple and handy, are often breached in praxis.

Since it was stated that communicational culture is a system of principles, values, and symbols that generate rules, rituals and institutions (here we used the term institution in the broadest sense possible, which encompasses not only the institutions of the state or public institutions, but also those of the civil society, such as marriage, godfathers, or matchmaking. When we referred to communicational culture, we talked about the institutions proper to communication – from social soirees to the spokesperson institution), we shall give some examples of such principles, values, and rules. 

Amongst the principles, the most general ones are the following (we shall enumerate only the general ones, which are considered to be universal in literature, for which reason some author also called them “laws”): the principle of cooperation, of relevance, of sincerity and of politeness (dealing tactfully with the interlocutor’s image). According to Gazdar and Levinson, the cooperative principle may be defined as it follows: “The cooperative principle makes our contribution such as it is required, at the stage at which it occurs, by the accepted purpose or direction of the talk exchange in which we are engaged” [**8**]. The observance of the cooperative principle does not entail the existence of an unshaded harmony between the participants to the verbal interaction; this principle highlighting the fact that, in order to render a possible communication, the existence of a minimum cooperation and of a common will of observing some rules is crucial. Regarding the principle of politeness, there is no “moral principle” telling us that being rude is not nice, but rather a communicational one that advices us that by observing it, we achieve an effective, more efficient communication. Whenever we threat the Other’s image, we lose him as our interlocutor; instead of focusing on the message, he will try to repair his image in our eyes, on building discursive strategies leading him to the fulfillment of this purpose.

Synthetically expressed, the maxims of an efficient communication are: the maxim of quantity (the speakers must convey the necessary information, no more and no less); the maxim of quality (the speakers must abide by reality, they shall not say things they believe to be false or for which they lack adequate evidence); the maxim of relation (the message conveyed by the speaker must be adequate to the purpose of communication and be linked to the interlocutors’ messages); the maxim of manner (the information must be significant for the context and the moment of the exchange); the maxim of style (the speakers must be clear, coherent, comprehensive, and concise); the maxim of receptivity – the speakers must adapt their messages to the traits of the recipients and to the knowledge they assumingly possess. 

After reviewing the principles and maxims of communicational culture, the identification of the values it comprises comes as a matter of fact, since they are derived from these principles and maxims: efficacy, efficiency, morality, cooperation, relevance, sincerity, politeness, truth, informativeness, empathy, clarity, coherence or concision. 

Furthermore, there is a series of requirements and conditions, which, once complied with, ensure an efficient communication: the conveyed information must be vivid, selective, adaptive and loyal, intelligible and accessible for the recipient; the messages must be conveyed as quickly as possible; a common language must be used; the lines of communication must be simplified and decongested through a decentralized adjustment of decisions to the inferior hierarchical layers; the sender and the receiver must be synchronized so as to avoid distortions; before trying to utter an idea, make it clear; the purpose of communication must be assessed and the text must be tailored to this purpose; the physical and human environment of the communication must be understood; in the planning phase, the opinions of the communication partners must be sought. 

**Typologies of patients; the differentiated communication with them**

In providing medical services, the odds of interacting with difficult personalities are high. We shall tackle here about one of the essential aspects of communication with patients: the identification of the types of difficult personality and the observance of some rules that derive from the particularity of this type. The patients facing a difficult situation may display extremely aggressive behaviors, due to the feelings and stress they are entangled in. Some of the approaches from the patient relations’ perspective that can be used during the interaction with difficult personalities will be discussed. Many papers and studies analyze human personality in view of offering ways of understanding different difficult personality typologies for a better communication with them. This is the reason why authors like B. Wang stressed the need for improving such a future communication between the doctor and his patients even since the doctor is a medical student [**13**]. Although they are not recipes that can be applied to the latter, since each personality is unique, their knowledge helps us in the communication process with difficult individuals. 

**Typology proposed by Karen Horney**

In a paper that became notorious, “Our inner conflicts” (1945), psychiatrist Karen Horney distinguished between three trends of showing sociability, whose knowledge is useful for the psychology of the relation with the public: a) moving toward people, b) moving against people; c) moving away from people [**6**].

a) The individuals from the first category have a powerful need of affection and approval, of finding a partner (friend, lover, husband/ wife) able to satisfy this need. They feel the need of being sought, wanted, loved, valued, of being accepted, close, appreciated, needed and even indispensable to the other; at the extreme, they feel the need of being protected or even guided. 

b) The individuals from the second category are tough and aggressive; they believe the world is an arena in which only the fittest are meant to survive; this leads to their primordial need of dominating and exploiting the others. They feel the need of being the best, of personal achievement, of enjoying prestige – regardless of the costs and means required for the fulfillment of these objectives.

c) The “detached” individuals have the inner need of setting an emotional distance between them and the others. The self-sufficiency and withdrawal satisfy their need of independence (not being influenced, constrained, obligated, and emotionally engaged). They do not stand conformity to the accepted behavioral norms. Loathing competitive fight, they live their lives as inconspicuous as possible. For such people, “l’infern c’est l’autres” (Jean-Paul Sartre).

According to Horney, whenever an individual looks at his/ her neighbor, he/ she may ask himself/ herself three categories of questions, depending on the category he/ she is part of: a)”Will he/ she like me?”; b) “How strong an adversary is he/ she?”; c) “Can he/ she be useful to me?” It is beyond doubt that in the social reality we shall not encounter these categories in their “pure” state. However, the combination between them may result in the domination of one of the types described by Horney. It is possible that an individual passes, throughout his life, from one dominance to another (the great stars of the political life, the sportsmen and the showmen often shift from (a) to (c) or from (b) to (a).

**Other categories of difficult personalities and ways of approaching them; a typology inspired from the medical practice**

1) Patients with dependent personality. The individuals with this type of personality rely on the others for all their needs, trying to select the people able to provide them with the emotional support they need and are willing to take decisions for them. Whenever they fail to obtain this support, they feel abandoned and become furious. They are terrified of the thought that they have to cope on their own and they are often so insistent that the ones around them reject them. Such persons habitually say, “You are the first person who has ever really understood me and listened to me. If you could spare more time, I would feel better”. If you spend more time with them than with the other patients, they will ask for more. If you interrupt the relation with them, they become furious, file complaints, or threaten to kill themselves (the last case is an extreme scenario). 

Recommended strategy. It is crucial that these persons are told that help will be provided, but within the time limit allotted for each patient. From the very beginning, they must be told that: “The 30 minutes allotted for our discussion are over. I would like to sum up what we have discussed and schedule our next appointment”. If the patient insists that he has something very important to add, he must be told that issue will be the first point on the agenda of the next meeting, should it still be a priority then. If patients try to contact you on the phone between appointments or to obtain additional appointments, you must refuse them categorically – excerpt for some particular circumstances. It is advisable that the doctor uses coaching techniques, helping patients formulate objectives and assume responsibility.

2) Histrionic patients. These are emotionally labile individuals, who suddenly shift their mood from happiness to sadness. As a rule, they are dramatic in presentation and seductive in the way they dress, talk, and move. In spite of these appearances, they are terrified with intimacy, and, consequently, they enter and exit various relationships with great celerity. If they feel that they intimacy is threatened during a discussion, meaning that their interlocutors ask them to go deep into exploring their feelings, they more away out of fear: they can become very nervous and leave the cabinet. During discussions, they often pay inappropriate compliments. Moreover, they make efforts to present themselves in the best light. 

Recommended strategy. It is crucial that any deviation from the rule is politely, but firmly, rejected: “I appreciate that you ask for my phone number, but I must emphasize that our relationship is a strictly professional one. I cannot be of any help otherwise.”

3) Narcissist patients. The persons displaying this type of personality need special attention. They do not tolerate a typical relationship and consider that any rule can be breached in order to serve their special needs. They become furious if treated as any other patient and if this happens, they threaten to file a complaint. Usually, the persons who interact with a narcissist individual have the tendency of changing the way in which they interact with them, paying a special attention to them. Their behavior is based on the fact that these individuals have a very low self-esteem. Their esteem is exclusively dependent on the extent to which they feel admired and respected by others. A detailed description of the trust-building strategies can be found in Nicolae-Iordache Iordache and Olivian Breda, who dedicated them an entire chapter in their book Who are You, Doctor? [**7**].

Recommended strategy. When such patients become furious, it is important to detect what exactly makes them angry and what would make them feel happier. It is possible to gratify them, without compromising the medical services. On the other hand, you must tell them that you cannot meet their needs, invoking practical arguments: “You insist to talk to our psychologist about your issues within maximum two weeks. Our psychologist, however, must make sure that he answers the needs of other patients as well. This is why we cannot schedule an appointment as frequently as you desire. However, we will do our best to schedule you as frequently as possible. Is this ok for you?” 

4. Obsessive-compulsive patients. These individuals are very concerned with morality and high standards. They have a strong consciousness but find it difficult to cope with feelings. Moreover, they find it difficult when their daily routine is threatened. Their high standards make them intolerant toward the behavior of others when they fall under these standards. They are obsessive with cleanliness and order. When in search for a job, the uncertainty troubles them, feeling that they lose control and they are afraid of a “psychic breakdown”. 

Recommended strategy. It is important to accept what the patient feels, to tell him that you understand his difficulty of coping with uncertainty. Whenever possible, review his evolution carefully and in exact terms. Their need for information should be established by asking them what exactly they want to find out: “I would like to know what exactly you expect from us”. Moreover, you should offer them more options of action, as much as possible, and ask for their opinion, or using the coaching techniques in order to help them identify the options on their own. If there is only one option available, you should explain them why.

5. “Borderline patients”. Certain individuals have experienced instable personal relationships, sudden changes of mood and impulsive behavior in the past. They often oscillate between intense affection and hatred toward people close to them. The same pattern will come up in the counseling relationship. They shift their disposition unexpectedly; have hard times coping with loneliness and with fulfilling tasks and give themselves over to impulses, regardless of the situation. They feel unloved and threatened by various people and circumstances. Paradoxically, their behavior makes the ones around them reject them, which they fear most. As such, you may deal with a patient who now admires you and then calls you names. These persons are not aware of their behavior and blame the others for what happens to them. 

Recommended strategy. You must remind yourselves that such persons fear relationships and expect to be disappointed. The patients who fall into this category have a continuous need of insurances. This is why it is important that you reassure them anytime possible, however, setting limits in dealing with their fears and concerns. If you work with other members of the team, this will help them treat them equally, not as good or bad. 

**A typology of the human personality in terms of temper; voluble persons and reticent persons**

1. Voluble persons. When voluble patients let themselves be carried away, you have to use techniques to silence them! The non-verbal language may be helpful. The change of the place or the turning over of the notes can make them realize that you need to move forward. You may choose a word and repeat it in order to change the subject: “fear … Oh, I understand, you felt fear. Tell me more about this.” 

Moreover, you can interrupt him and explain that the following question is important and that you have to address it. You must be assertive, but not aggressive: “I am sorry to interrupt you, but it is important for me to understand the succession of the events. Have you been turned down after the interview?” Alternatively, you may interrupt him as it follows: “I understand that this thing bothered you, but we do not have much time left. Can we come back to this later?” It is important that you give the meeting a pre-established structure, a framework, and time. “This is what I would like us to do: in the following 10 minutes I want to find out what happened to you in the accident yesterday and then I would like to create an action plan. Do you agree?” 

Used carefully, some of the following phrases may determine the talkative patient remain focused on the issue: “Yes, I am sorry that you experienced that, but could we go back to…?”; “I think we worn this topic out, let’s shift to …” (this approach indicates that the information supplied by the patient is not useful); “Did you finish what you had to say? (this question implies an “Yes” or that you consider the topic closed). 

2. Reticent persons. Obtaining necessary information from shy or reticent persons requires time, patience and a different approach. The tone has to be low and unprovocative. The aim is to be in line with the patient’s style and disposition. Finding out the problems that retractile persons face can only occur in a context based on trust. You may find out more relevant information on their situation by using the active listening techniques. The body language and the tone of the voice will indicate your interest. 

The shy patient must be carefully led throughout a discussion. When his emotions are obvious, he must be asked: “Are you all right? May I ask you what bothers you?”. You must use any information the patient offers and require clarifications: “You seem solicitous about this situation and you don’t want to talk too much about it. Could you tell me what you believe it could be?” 

There are situations when the patient remains inaccessible and it is hard to find out more from him. In this case, you have to acknowledge the difficulty of having a dialogue with the patient: “I would like to know if you have problems in which I could help you. However, it seems that we face some difficulties in our conversation. ”This way, you highlight that the problem between the two is due exclusively to the patient.” 

Some persons are, by their nature, quiet or shy and it is more difficult to gain their trust. There are also some temporary factors that trigger such a withdrawal. In order to identify the reasons of this reaction, it is important to find out whether the patient is willing to reveal his difficulties: “Could you tell me why you find it so hard to talk to me right now?” Some severe withdrawal manifestations are caused by depression or other psychical disorders; in this case, the advice of a specialist being required. 

**Difficult personalities due to the needs of control, perfection, approval and attention**

Another classification of difficult people was developed by Bramson (1981) [**4**], enhanced by Brinkaman and Kirschner (1994) [**5**], who postulated the existence of the high needs for control, perfection, approval and attention, needs that form the basis of difficult personalities. 

People with high needs for control are obsessed with completing the task and take pride in getting a job done quickly. 

**Table 1:** People with high needs for control

The “Tank”	Gets things done quickly by giving orders, being pushy, yelling and at times being too aggressive
The “Sniper”	Controls people by using sarcasm, embarrassment and humiliation
The “Knot-It-All”	Dominates conversations, not listening to the others’ ideas and rejecting the arguments counter to his position

People with high needs for perfection are obsessed with completing a task correctly. 

**Table 2:** People with high needs for perfection

The “Whiner”	Constantly complains about the situation but never tries to change it
“No person”	Believes that nothing will ever work and disagrees with every suggestion or idea
“Nothing Person”	Responds to difficult situations by doing and saying nothing – he simply gives up and retreats

People with high needs for attention are obsessed with being appreciated. When they do not feel appreciated they have different negative reactions. 

**Table 3:** People with high needs for attention

The “Grenade”	(equivalent of the hysterical) – he yells.
The “Friendly sniper”	Gets attention by poking fun at others
The “Think-They-Know-It-All”	Exaggerates, lies and gives unwanted advice to get attention

People with high needs for approval are obsessed with being liked. Their behavior is often centered on gaining approval rather than completing a task correctly and quickly. 

**Table 4:** People with high needs for approval

“Yes Person”	Agrees with everything and, as a result, often agrees to do so much that he cannot honor his commitments. He is afraid of giving up part his tasks because he fears he will be judged by the others.
“Maybe Person”	Avoids conflicts by never taking a stand in any issue

If we have mentioned above the behaviors of difficult people, with low self-esteem and high need for control, Raynes (1997) [**10**] indicated a more profound relation of such behaviors of personality: the high self-esteem and confidence can be correlated with the behavior of the “Think-They-Know-It-All” and “No person”; the personalities variable of extroversion were correlated with gossiping and a high level of work interest was positively correlated with the behavior associated with the “Yes Person” and negatively correlated with the “Whiner” (Raynes, 1997) [**10**]. Of course, these typologies are extreme. In conflict situations, the use of the recommended strategies may alleviate emotions and difficult behaviors. However, in special cases, it is advisable to consult a specialized psychologist or psychiatrist. Facing such situations can be exhausting. These are just a few landmarks that might help you build customized and patient-oriented communication relations. When you interact with patients displaying difficult behaviors, apart from the presented strategies, we must take into account Bramson’s recommendations (Bramson, 1981) [**4**]: do not let yourself led by emotions, do not say “I am sorry” too often, do not take anything personally, build the relationship!

Words that can or may irritate: “This is our policy”, “You cannot …”; “You should……”; “I cannot help you”; “All we can do is…”; “I have nothing to do with this problem”; “I am new here”. Behaviors that can or may irritate: apathy; transfer of the problem to others; hiding behind policies and procedures; arrogance; lack of respect; aggressiveness; wrong information; failure of undertaking responsibilities and aggressiveness (physical but particularly symbolical). As shown elsewhere [**3**], in communication symbolic thinking is as important as rational thinking. What patients expect from the medical professionals: to be treated with dignity and respect, flexibility; quality; settlement of issues, responsibility; support. 

Finally, it has to be highlighted that all these considerations are submitted to the borders generated by an ethical approach of communication. These borders always help us distinguish between persuasion and manipulation [**11**,**12**]. 
